# The Electrical Exhibition

**Published:** 1905-10-07

**Authors:** 


### THE ELECTRICAL EXHIBITION.

The Electrical Exhibition at Olympia is both interesting and instructive. A good many of the exhibits are directly applicable to medical and institutional use, apart from those connected with lighting and heating, both of which are, of course, especially suitable where sick people are housed. Proceeding down one of the side avenues of the Exhibition we became conscious of a pungent smell in the atmosphere. When supplied a few minutes later with the literature supplied by the Ozonair Company we read : "It has a most refreshing smell, comparable only to the bracing air felt at the seaside or on mountain country." On this point we reserve our opinion, but as Ozonair also claims to be a most powerful disinfectant it merits our attention. Ozonair is said to be a chemically-produced ozone diffused in the air by an apparatus somewhat of the appearance of the gramophone. It is especially recommended for use in hospitals and all buildings likely to be vitiated by unhealthy surroundings or many occupants. In the prospectus a large number of eminent scientific men are quoted to establish the claims of ozone as a hygienic force, and the price of the ozone to the public does not exceed, we are told, one six-hundredth part of a penny for every 1,000 cubic feet, whilst the installation itself is not costly. The London depot is at 27 Chancery Lane.

In another part of the building an electric table humidifier is to be found, manufactured by James Keith, of 27 Farringdon Avenue. The idea is, of course, to provide a simple and ornamental means of moistening the air or introducing some medicinal preparation into it. The humidifier can be introduced where electric light is installed. The spray which it diffuses is so fine as to be almost invisible, and as much as 12 pints of liquid can be distributed in the hour. It is also claimed for it that it is useful for ventilating purposes, as it is capable of charging 26,250 cubic feet of air per hour.

Darton and Co., of 142 St. John Street, E.C., have an interesting stall of novelties at the Exhibition. Amongst these are some very inexpensive table and wall ventilating motor-fans, and also some magneto-electric medical machines. To those who desire to exhibit or use electric models the very varied collection of electric motor models will be found most useful.

At other stalls will be found many contrivances which are of interest to medical men.

